# From Psoriatic Arthritis to Takayasu Arteritis and Stroke: A Challenging Journey of Overlapping Autoimmune Rheumatic Diseases in a Young Female

**DOI:** 10.7759/cureus.69673

**Published:** 2024-09-18

**Authors:** Abraham Mohan, Anuradha Ramachandran, Joseph Sebastian

**Affiliations:** 1 Rheumatology, Caritas Hospital, Kottayam, IND; 2 Radiology, Caritas Hospital, Kottayam, IND; 3 Neurology, Caritas Hospital, Kottayam, IND

**Keywords:** inflammatory arthritis, large vessel vasculitis, psoriatic arthritis, stroke, takayasu arteritis

## Abstract

This case report details the clinical presentation, diagnosis, and management of a 23-year-old female with a unique medical history, including psoriatic arthritis (PsA) and Takayasu arteritis (TAK) and ultimately presenting with acute ischemic young stroke. The patient initially presented with right hip and buttocks pain, multiple itchy skin lesions, and right sacroiliac joint pain in 2017. She was diagnosed with psoriatic arthritis and treated effectively, achieving complete remission by 2020. In August 2023, she presented with acute-onset neurological deficits and vascular symptoms leading to a diagnosis of TAK. This case highlights the challenges in diagnosing and managing a complicated case with overlapping autoimmune rheumatic diseases.

## Introduction

Takayasu arteritis (TAK) is a chronic granulomatous large vessel vasculitis that affects the aorta, its main branches, and pulmonary arteries. The inflammatory process initially leads to thickening of the arterial wall and may result in stenosis, occlusion, dilatation, or aneurysm formation [1,2]. It predominantly occurs in young women and can lead to significant morbidity due to vascular occlusion and resultant ischemia [3]. The pathophysiology of psoriatic arthritis (PsA) is characterized by interplay of genetic, environmental, and immunological factors. Psoriatic arthritis, a chronic inflammatory arthritis associated with psoriasis, can also cause significant joint damage and disability [4]. The co-occurrence of these two conditions presents a unique clinical challenge and is a rare scenario. The stated prevalence of TAK ranges from 4.7 to 360 occurrences per million [5]. Among these, PsA and TAK are notable for their distinct pathological features and significant clinical impacts. PsA is a chronic inflammatory arthritis associated with psoriasis, and TAK is a rare, large vessel vasculitis that primarily affects young women [3]. This case report discusses the clinical presentation, diagnosis, and management of a young lady who developed Takayasu arteritis after achieving remission from psoriatic arthritis.

## Case presentation

**Figure 1:**
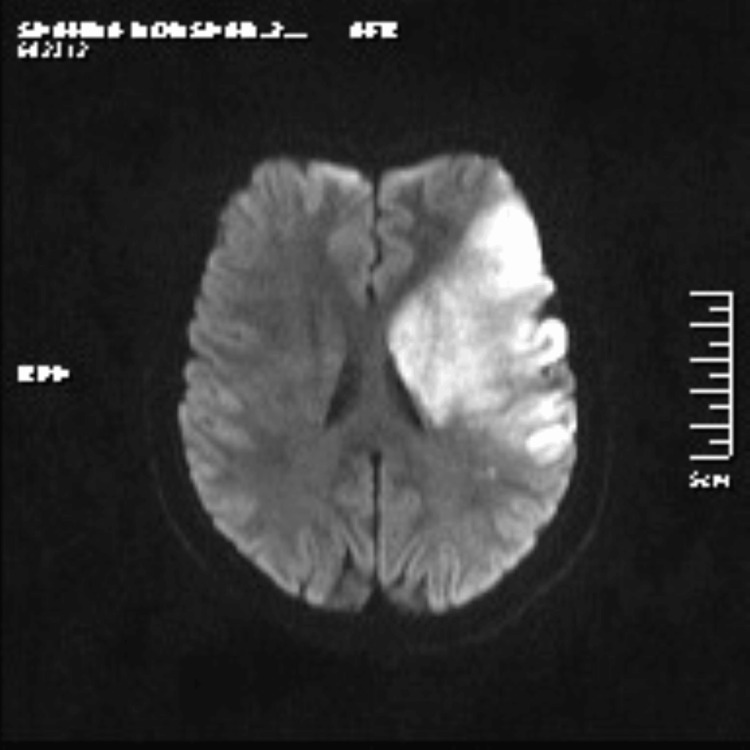
MRI brain showing acute infarct in the left fronto-parieto-temporal lobe and left capsulo-ganglionic region Moderate-sized acute infarct in the left middle cerebral artery territory involving left fronto-parieto-temporal lobe and left capsulo-ganglionic region. Mild mass effect is seen in the form of effacement of cortical sulcal spaces and mild compression of left lateral ventricle.

**Figure 2:**
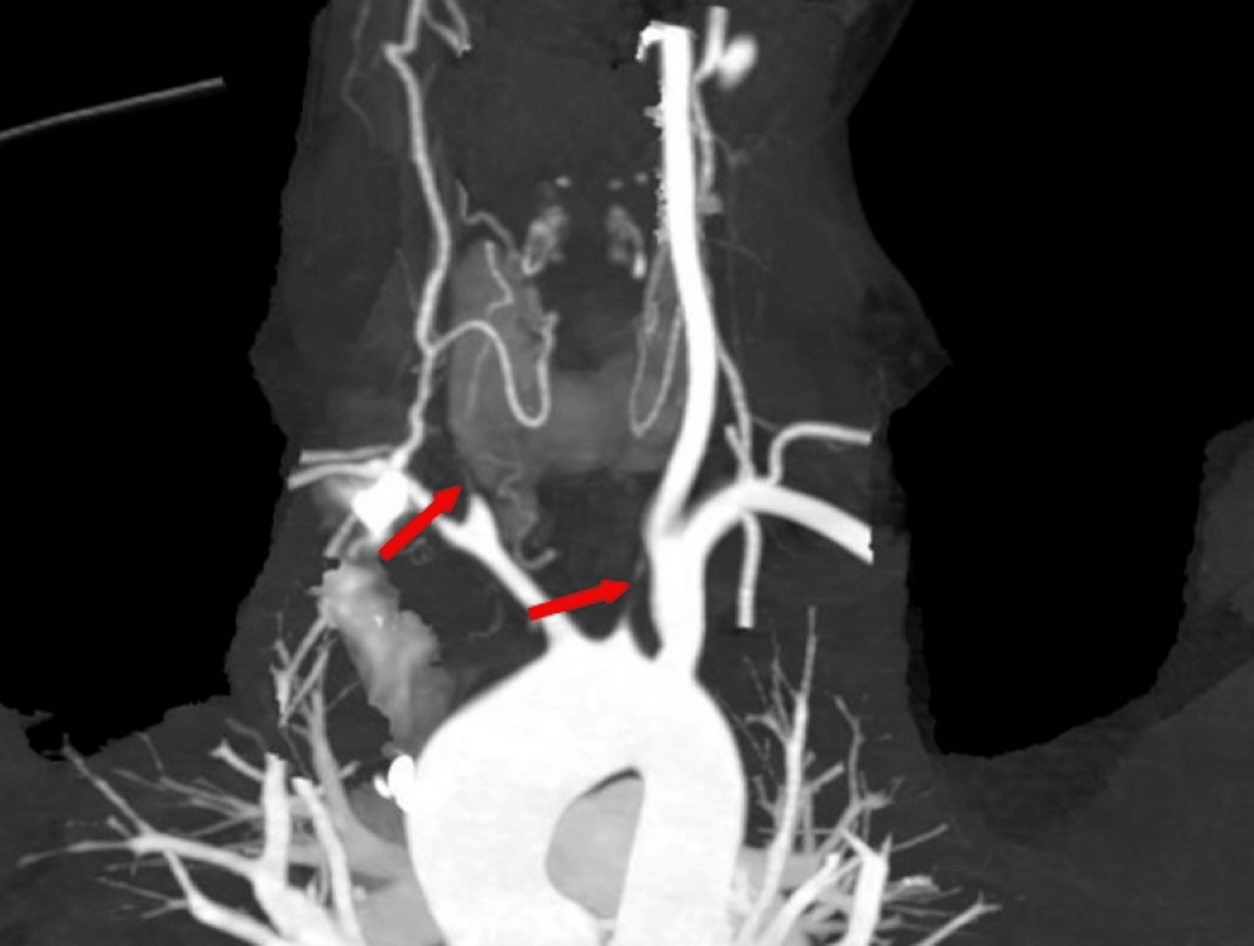
CT angiogram image showing bilateral common carotid artery (left and right CCA) stenosis with occlusion CT angiogram image shows the major vascular structures in the neck and upper thoracic region. The aortic arch is prominently seen at the bottom of the image with subtle diffuse arterial wall thickening of the proximal aortic arch. Minimal thickening is noted at origin of the left subclavian artery followed by normal caliber of rest of the artery and left vertebral artery. There is significant circumferential wall thickening of the left common carotid artery producing multifocal occlusion in proximal half. There is significant luminal narrowing (>50%) noted in distal half of left common carotid artery (arrow) up to bifurcation due to thrombus formation. The right common carotid artery shows near-complete occlusion throughout its length, while the innominate artery and right subclavian artery exhibit over 50% narrowing (arrow). The proximal right vertebral artery is not visualized with distal segment showing slightly reduced caliber and normal contrast enhancement. Mild thickening of the left common femoral artery and enlarged left external iliac nodes was also noted.

A 17-year-old female first presented in 2017 with a 1.5-month history of right hip and buttocks pain radiating to the legs and multiple itchy skin lesions. On examination, she had tenderness over the right sacroiliac joint and discrete scaly plaques on the left foot and knee. MRI was suggestive of right sacroiliitis, and a skin biopsy done showed psoriasiform dermatitis. She was diagnosed with psoriatic arthritis and treated with multiple disease-modifying anti-rheumatic drugs over the course of three years which included low-dose methotrexate, tofacitinib, and sulfasalazine. By 2020, she was in complete remission and off medications. In August 2023, she presented with a history of acute-onset giddiness followed by loss of consciousness with frothing from the mouth and right-sided weakness on the last day. On examination, her right radial, brachial, and bilateral common carotid artery pulses were not palpable, while all other peripheral pulses were felt. Blood pressure in the left arm was 120/70 mm Hg, and it was not recordable in the right arm. Muscle power was grade 1 in the right upper limb and grade 3 in the right lower limb, with brisk reflexes and a positive Babinski sign on the right side. She had right-sided hemiplegia with right upper motor neuron facial nerve palsy. MRI of the brain showed a moderate-sized acute infarct in the left middle cerebral artery territory involving the left fronto-parieto-temporal lobe (Figure 1). MR angiography of neck and brain vessels revealed abrupt cutoff of bilateral common carotid arteries (CCAs) at the origin, with near-complete non-opacification of bilateral external carotid arteries (ECAs) and bilateral internal carotid arteries (ICAs). There was abrupt cutoff of proximal M1 segment of the left middle cerebral artery (MCA) with complete non-opacification of entire left MCA. Right MCA and bilateral anterior cerebral arteries were reformed from posterior circulation through right posterior communicating artery. There was significant focal luminal narrowing (70%-80%) at the origin of the right vertebral artery (VA). She was started on antiplatelets and a cardiology evaluation, including that 2D echocardiography was normal. She was not considered a suitable candidate for thrombolysis or mechanical thrombectomy as 24 hours had elapsed since the onset of her symptoms. Large vessel vasculitis was suspected as the cause of young stroke as she had probable bilateral CCA stenosis with right VA stenosis. Further investigations to rule out other secondary causes were negative including antinuclear antibody, Venereal Disease Research Laboratory (VDRL) test, homocysteine levels, and antiphospholipid antibodies. A computed tomography aortogram revealed wall thickening of the aortic arch, bilateral CCA, bilateral subclavian arteries, innominate artery, and right VA suggestive of arteritis consistent with Takayasu's arteritis type II (Figure 2). The arterial wall thickening mentioned above did not show differential wall enhancement. An intraluminal thrombus producing significant luminal narrowing of the left CCA (more than 50%) was noted in the distal half up to the bifurcation. She was started on intravenous steroids (dexamethasone 4 mg 8th hourly), with symptomatic improvement. She was later switched to oral steroids and mycophenolate. Her muscle power improved in the right upper and lower limb, and she was able to walk with support, but she developed intermittent dizziness probably due to bilateral CCA occlusion. She was referred to a higher center for ECA-ICA surgical bypass to increase blood flow to the brain and relieve symptoms of cerebrovascular ischemia but was deemed to be an unsuitable candidate for the procedure.

## Discussion

Takayasu arteritis is a global condition, but it is more prevalent in Asian countries, particularly affecting women more than men, with onset typically between the ages of 10 and 40. This aligns with our case, involving a young South Asian woman who began experiencing symptoms in her second decade of life [6,7]. This case is an example of an unusual overlap of autoimmune rheumatic diseases (ARDs). This case shows the challenges in diagnosing and managing a patient who developed a second autoimmune rheumatic disease, which was further complicated by the development of an acute ischemic stroke. Takayasu's arteritis is an idiopathic progressive inflammatory disease of the aorta and its branch arteries. Psoriatic arthritis is a progressive, chronic, autoimmune inflammatory disease that is associated with psoriasis. The clinical manifestations of PsA are complex and heterogeneous [8,9]. Takayasu's arteritis is simplified by classification into three stages for the purpose of understanding as types I, II, and III. Type III is the burnt-out fibrotic stage, which is characterized by minimal symptoms and remission of the disease. In the presented case, the initial symptoms of loss of consciousness and frothy mouth were atypical for Takayasu arteritis. Most clinical literature describes more common presentations related to vascular insufficiency or systemic inflammation, such as claudication, hypertension or systemic symptoms like fever and weight loss [10]. Different symptoms have been reported as the initial symptoms of the disease. Backache is rarely described in the medical literature as the presenting symptom. Diagnosing Takayasu's arteritis can be challenging due to its nonspecific symptoms and overlapping features with other vasculitides and autoimmune conditions [2,10,11]. Psoriatic arthritis and Takayasu arteritis both involve significant inflammatory processes, but they affect different tissues and organs. The transition from remission of psoriatic arthritis to the acute presentation of Takayasu arteritis shows the need for monitoring and comprehensive evaluation when new symptoms arise. There may be shared immunopathogenic pathways or triggering factors that contribute to the development of both conditions. Strokes are a common consequence of Takayasu's arteritis, with a 10% to 20% prevalence [12]. Stroke as the initial manifestation of Takayasu arteritis is rare, with only a few cases reported in the literature [13,14]. When arteries supplying the brain such as the carotid or vertebral arteries become severely narrowed or obstructed, it can result in reduced blood flow, increasing the risk of ischemic stroke. Inflammation within the blood vessels can also contribute to clot formation, further heightening the risk of stroke. Active Takayasu's arteritis rather than psoriatic arthritis has contributed to the acute ischemic stroke in this patient. Embolism from thrombus in the left CCA or inflammatory activity due to Takayasu arteritis must have contributed to the left MCA occlusion in this patient, resulting in stroke. In addition to the increased risk caused by elevated cardiovascular risk factors, population studies have shown that psoriasis independently contributes to an increased risk of myocardial infarction or stroke. In both conditions Takayasu's arteritis and psoriatic arthritis, the chronic inflammation and potential damage to the vasculature can predispose individuals to stroke [15]. In this case, CT angiography played a crucial role in identifying the characteristic manifestations of Takayasu's arteritis [16-18]. Diagnostic imaging like the computed tomography aortogram (CTA), magnetic resonance angiogram (MRA) or positron emission tomography-computed tomography (PET-CT) scan are fundamental to diagnosis and disease monitoring [19-21]. Fluorodeoxyglucose (FDG) PET-CT allows for earlier diagnosis, which reduces the need for additional needless tests and hospitalization costs. PET-CT helps by detecting areas of active inflammation in the arterial walls, which is crucial in the early stages of the disease when structural changes may not yet be visible on traditional imaging methods. The uptake of a radiotracer, such as fluorodeoxyglucose (FDG), highlights metabolically active inflammatory cells, allowing clinicians to assess the extent and severity of the disease [22,23].

Studies have stated that Takayasu's arteritis does co-occur with inflammatory bowel disease (IBD), ankylosing spondylitis (AS) or Behcet's syndrome (BS), and other various inflammatory disorders [24]. Another study also stated that spondyloarthropathies (SpAs) are common in patients with Takayasu arteritis suggesting shared pathogenetic mechanisms [25]. In this case, the patient's initial presentation with psoriatic arthritis was followed by a period of remission, to the development of Takayasu's arteritis. This sequence underscores the importance of continuous monitoring and evaluation when new symptoms arise, particularly in patients with a known history of an ARD.

## Conclusions

This case highlights the importance of considering large vessel vasculitis in young patients presenting with unexplained neurological and vascular symptoms in the context of a known ARD such as PsA. PsA was in remission in this patient, making it the unlikely cause of stroke. Active Takayasu arteritis led to stroke in this patient. This patient was brought to our center 24 hours after onset of symptoms, hence could not be thrombolyzed or undergo mechanical thrombectomy which could have reduced the impact of stroke. Comprehensive clinical evaluation, appropriate use of imaging modalities, and timely initiation of stroke management and immunosuppressive therapy is imperative in such cases. This case serves as a reminder of the challenges in diagnosing and managing complicated ARDs and underscores the importance of a multidisciplinary approach in providing optimal care for these patients.
